# High-resolution PGT-A results in incidental identification of patients with small pathogenic copy number variants

**DOI:** 10.1007/s10815-023-02969-8

**Published:** 2023-11-14

**Authors:** Deirdre Leahy, Diego Marin, Jia Xu, Jennifer Eccles, Nathan R. Treff

**Affiliations:** Genomic Prediction Inc., 671 US Highway One, North Brunswick, NJ 08902 USA

**Keywords:** Preimplantation genetic testing, Reciprocal translocation, Copy number variant, In vitro fertilization

## Abstract

**Purpose:**

This study aimed to evaluate whether a high-throughput high-resolution PGT-A method can detect copy number variants (CNVs) that could have clinical implications for patients and their embryos.

**Methods:**

A prospective analysis of PGT-A cases was conducted using a high-resolution SNP microarray platform with over 820,000 probes. Cases where multiple embryos possessed the same segmental imbalance were identified, and preliminary PGT-A reports were issued recommending either parental microarray or conventional karyotyping to identify CNVs or translocations.

**Results:**

Analysis of 6080 sequential PGT-A cases led to identification of 41 cases in which incidental findings were observed (0.7%) and parental testing was recommended. All cases, in which parental studies were completed, confirmed the original PGT-A incidental findings. In 2 of the cases, parental studies indicated a pathogenic variant with clinical implications for the associated embryos. In one of these cases, the patient was identified as a carrier of a duplication in chromosome 15q11.2:q11.2 (SNRPN + +), which is associated with autism spectrum disorder. In the second case, the patient was heterozygous positive for an interstitial deletion of 3p26.1:p26.3, which is associated with 3p deletion syndrome and had clinical implications for the patient and associated embryos. In each case, parental studies were concordant with PGT-A findings and revealed the presence of an otherwise unknown CNV.

**Conclusion:**

High-throughput high-resolution SNP array–based PGT-A has the ability to detect previously unknown and clinically significant parental deletions, duplications, and translocations. The use of cost-effective SNP array–based PGT-A methods may improve the effectiveness of PGT by identifying and preventing previously unknown pathogenic CNVs in children born to patients seeking in vitro fertilization.

**Supplementary Information:**

The online version contains supplementary material available at 10.1007/s10815-023-02969-8.

## Introduction

Preimplantation genetic testing (PGT) specifically for aneuploidies (PGT-A) has increased in vitro fertilization (IVF) efficiency by enabling the selection of embryos not deemed to carry chromosome aberrations, which are often incompatible with life, and thus increases implantation success and reduces miscarriages rates [1, 2]. PGT technologies have expanded beyond screening for the common aneuploidies and are currently being implemented to address the risk for inherited genetic changes such as structural rearrangements via PGT-SR, monogenic conditions and small deletions/duplications via PGT-M, and polygenic disorders via PGT-P. Prior studies have demonstrated the potential to reveal previously unknown parental findings via PGT-A in trophectoderm biopsies. For example, it has been demonstrated that recurrent segmental imbalances seen in multiple embryos from the same patient can lead to the identification of balanced translocation carrier parents [3–5].

Contemporary methods of PGT-A involve the use of technologies that provide higher resolution that may uncover not only translocation carrier parents but also carriers of smaller and potentially pathogenic imbalances such as copy number variants (CNVs). There are various PGT-A technologies that result in differing levels of resolution and limitations. In the context of PGT-A, next-generation sequencing (NGS) [6] methods are applied using a “lowpass” approach which in turn provides low genomic resolution and may miss clinically relevant copy number variants (CNVs). Alternative methods, such as high-throughput high-resolution single-nucleotide polymorphism (SNP) array–based PGT-A, may be capable of detecting pathogenic CNVs. Contemporary SNP array technology allows 96 samples to be analyzed in parallel in conventional 96-well plates, with each array providing data on over 800,000 positions in the genome. This results in significantly higher resolution compared to conventional low-pass NGS PGT-A methods.

This capability may be important given that the incidence of a microdeletion or microduplication syndrome in pregnancy is 1.6%, and subsequent parental studies revealed that risk of an inherited pathogenic copy number variant is approximately 34.4% [7]. It has also been established that CNVs are a risk factor for different neuropsychiatric disorders including but not limited to autism [8]. Studies have shown that both de novo and inherited CNVs play a role in an individual’s susceptibility of various neurodevelopmental disorders and some families with a history of autism spectrum disorders have been found to have a higher CNV burden [9]. The ability to detect the risk for CNVs via PGT technologies would provide more accurate classification, clinical management, and selection of suitable embryos. Our data indicates about 0.7% incidence of detection across all embryos analyzed. This reduction compared to the 1.6% incidence of a microdeletion or duplication syndrome in pregnancy is expected as these are often de novo in origin, whereas the incidental findings we reported on were confirmed to be parental in origin. Additionally, these findings were not previously known, prior to PGT-A studies. If a hereditary microdeletion or microduplication syndrome is known, targeted PGT-M studies would be indicated thus explaining the differences in incidence. However, this study succeeds in demonstrating the potential to accurately detect previously unknown small copy number changes and provides evidence that this technology could be used to detect de novo microdeletion and microduplication syndromes.

## Materials and methods

Routine PGT-A using high throughput SNP array technology was performed at a single PGT reference laboratory. This SNP array platform provides high resolution with over 820,000 probes per embryo biopsy, and was extensively validated on positive controls as previously described [10]. All PGT-A was performed on trophectoderm biopsies. In cases where there were multiple embryos with the same segmental imbalance, specifically the same small deletion, duplication, or segmental imbalance in two or more embryos, follow-up genetic testing on the parents was recommended.

In cases where a single interstitial imbalance (CNV) was detected in multiple embryos, microarray testing was recommended. In cases where two different chromosomes displayed telomeric imbalances, conventional g-banding karyotyping was recommended. All cases where parental microarray and karyotype reports were obtained were reviewed for concordance with the predictions made from PGT-A. When PGT-A results were confirmed, preliminary PGT-A reports were amended to include inheritance of parental imbalances.

## Results

### Identification of translocation carriers

**Fig. 1:**
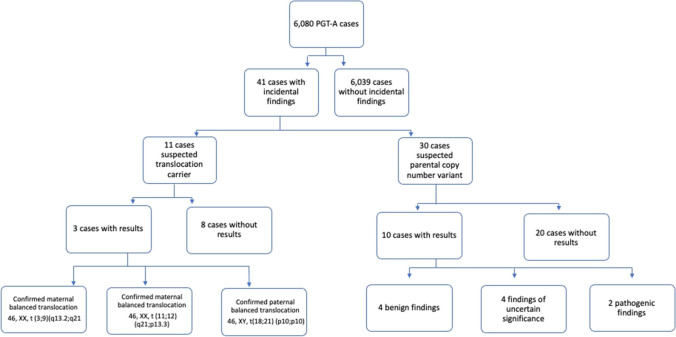
Tree diagram illustrating identification and follow-up on cases with incidental findings for possible translocation or CNV

**Fig. 2:**
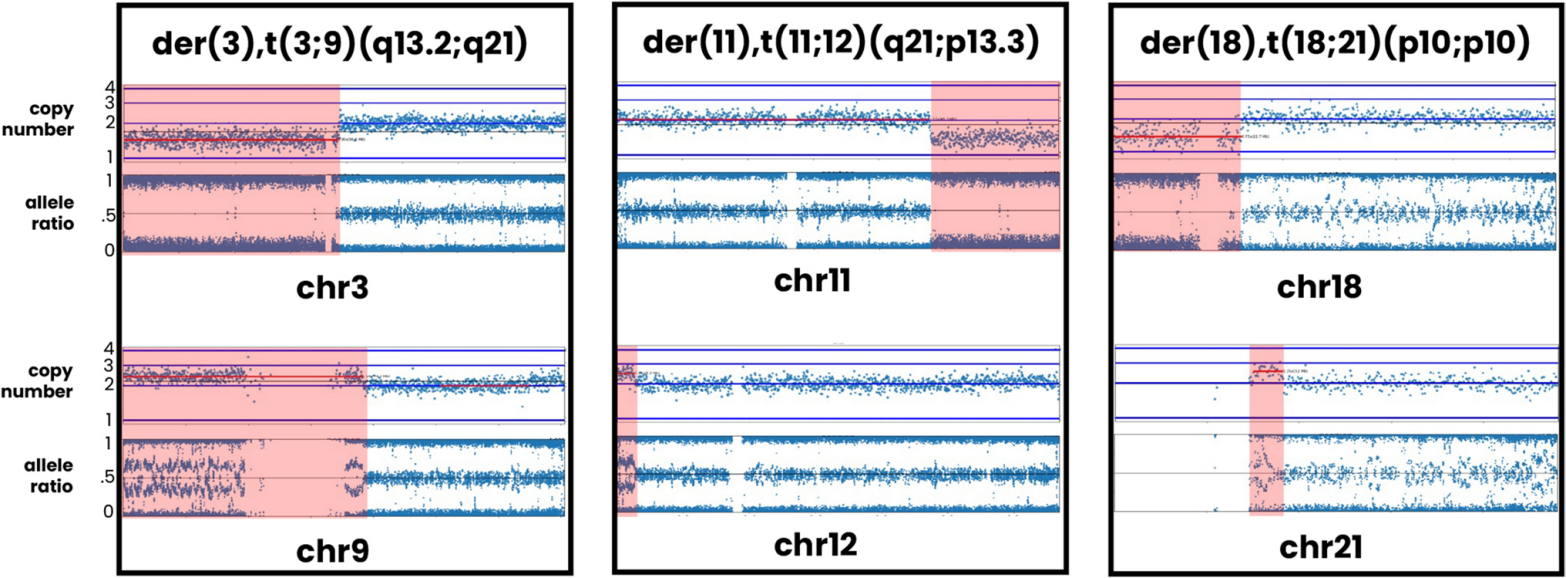
Examples of SNP array PGT-A results for 3 cases leading to identification of parental carriers of a balanced translocation. In each case, two chromosomes displayed clear copy number and allele ratio imbalances (highlighted in red). Although each case displays results observed in only one embryo, multiple embryos possessed imbalances on the same chromosomes

Overall, there were 41 cases in which incidental findings were observed and parental testing was recommended. Results of parental testing were obtained in 13 of the 41 cases, and all results confirmed the original PGT-A predictions. In cases where the parental reports were not received, it is unknown if testing was ordered. If so the outcome of any potential testing was not reported to us by the referring clinician. In 3 cases, a balanced translocation carrier status was confirmed by conventional karyotyping, supporting IVF-derived embryo-based PGT-A incidental findings (Figs. 1 and 2; Table 1).

#### Case 1

The patient was 44 years old at the time of egg retrieval. Five trophectoderm biopsy samples were received for PGT-A. After SNP microarray analysis, 4 embryos were predicted to be aneuploid. Two of the embryos had consistent segmental imbalances on chromosomes 3 and 9. A conventional karyotype of the parents was ordered and confirmed a maternal balanced translocation, 46,XX,t(3;9)(q13.2;q21). Once a balanced translocation was documented on conventional karyotype, the PGT-A results were reinterpreted using a previously established PGT-SR approach. After re-analysis, one embryo was predicted to be a balanced translocation carrier, 2 embryos were unbalanced, and the remaining two embryos were balanced carriers but had additional chromosome abnormalities unrelated to the maternal translocation, and therefore were predicted aneuploid.

#### Case 2

The patient was 34 years old at the time of egg retrieval. Nine trophectoderm biopsy samples were received for PGT-A. After SNP microarray analysis, 6 embryos were predicted to be aneuploid. Three of the embryos had consistent segmental imbalances on chromosomes 11 and 12. A conventional karyotype of the parents was ordered and confirmed a maternal balanced translocation, 46,XX,t(11;12)(q21;p13.3). Once a balanced translocation was documented on conventional karyotype, the PGT-A results were reinterpreted using a previously established PGT-SR analysis protocol. After re-analysis, three embryos were predicted to be balanced translocation carriers, 2 embryos were predicted to be unbalanced, but due to additional chromosome abnormalities unrelated to the maternal translocation were predicted to be aneuploid. The remaining 5 embryos were identified as aneuploid due to chromosome abnormalities unrelated to the maternal translocation.

#### Case 3

The patient was 27 years old at the time of egg retrieval. Six trophectoderm biopsy samples were received for PGT-A. After SNP microarray analysis, 4 embryos were predicted to be aneuploid. Three of the embryos had consistent segmental imbalances on chromosomes 18 and 21. A conventional karyotype of the parents was ordered and confirmed a paternal balanced translocation, 46,XY,t(18;21)(p10;p10). Once a balanced translocation was documented on conventional karyotype, the PGT-A results were reinterpreted using a previously established PGT-SR analysis protocol. After re-analysis, two embryos were predicted to be euploid, and 2 embryos were predicted to be unbalanced. One embryo was predicted to be unbalanced but was identified as aneuploid due to additional chromosome abnormalities unrelated to the paternal translocation. The remaining embryo was identified as aneuploid due to chromosome abnormalities unrelated to the paternal translocation.

### Identification of pathogenic CNV carriers

CNV carrier status was confirmed in all 10 cases where parental microarray or karyotype data was obtained (Table 1). In 4 of the cases, parental studies indicated a benign CNV and the embryo PGT-A results were reinterpreted accordingly. In 4 of the cases, parental studies indicated a CNV classified as a variant of uncertain significance and the embryo.

**Fig. 3:**
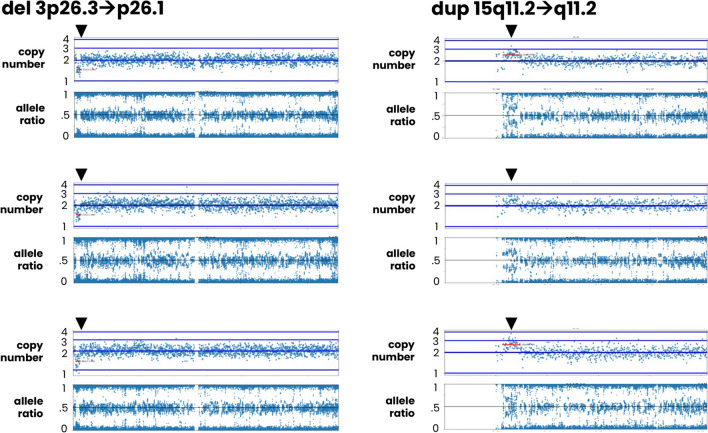
Examples of SNP array PGT-A results from 2 cases leading to the identification of parental carriers of a pathogenic CNV. Each case displays results from 3 embryos where a small imbalance was clearly seen (highlighted in red). The del 3p26.3:p26.1 was 3 Mb, and the dup 15q11.2:11.2 was 6.5 Mb

PGT-A results were reinterpreted accordingly. In 2 of the cases, parental studies indicated a pathogenic variant with clinical implications for the associated embryos (Fig. 3) and are described in detail below.

#### Case 1

The patient was 41 years old at the time of egg retrieval. Five trophectoderm biopsy samples were received for PGT-A studies. After SNP microarray analysis, all 5 of the embryos were predicted to be aneuploid. Three of the embryos had a recurrent segmental imbalance on chromosome 15. A conventional karyotype of the parents was ordered and confirmed an abnormal female karyotype with a duplication within 15q11.2. When maternally inherited, this duplication is associated with autism spectrum disorders and had clinical implications for the patient and associated embryos. PGT-M studies were recommended but not completed.

Based on the preliminary results and maternal karyotype. all the embryos were aneuploid.

#### Case 2

The patient was 27 years old at the time of egg retrieval. Four trophectoderm biopsy samples were received for PGT-A studies. After SNP microarray analysis, all 4 of the embryos were predicted to be aneuploid. Three of the embryos had a recurrent segmental imbalance on chromosome 3. A chromosome microarray of the parents was ordered and confirmed that the patient was heterozygous positive for an interstitial deletion of 3p26.1:p26.3, which is classified as pathogenic and associated with 3p deletion syndrome having clinical implications for the patient and associated embryos. PGT-M studies were recommended but not completed. Based on the preliminary results and maternal karyotype, all the embryos were aneuploid.

## Discussion

This study has demonstrated that a high-throughput high-resolution SNP array PGT-A platform has the ability to detect previously unknown and clinically significant parental CNVs, such as microdeletions, microduplications, and translocations. The identification and confirmation of the parental findings in these cases provided additional clinical information such as the reason for reproductive failure, personal diagnosis, and may have averted the delivery of an affected child. In each case, parental studies were concordant with embryo PGT-A findings and revealed the presence of an otherwise unknown CNV. SNP array technology provides an alternative method with 80 times the resolution of conventional NGS methods used in a PGT-A setting with the smallest parental CNV identified in this study being 914.7 Kb. SNP array technology has increased throughput capability while also significantly reducing the cost per sample. The use of cost-effective high-resolution SNP array–based PGT-A methods may improve the effectiveness of PGT-A by identifying and preventing previously unknown pathogenic CNVs in children born to patients seeking IVF. Although conventional NGS may be able to detect microdeletion/duplication syndromes, low-pass NGS methods provide low genomic resolution which may miss clinically relevant copy number variants (CNVs) and this study demonstrated the novel clinical applications a high throughput PGT-A outside of routine chromosome analysis. It may be useful to establish criteria for recommending parental testing based on observations made in embryos as demonstrated in this case report series.

The ability to identify patients with translocations or small deletions and duplications may be impaired by having too few embryos to observe more than one embryo with the same imbalance. The minimal incidence of pathological CNV findings in comparison to aneuploidies is significant; however, we argue that any pathogenic finding that impacts embryo health and selection is relevant and the ability to expand routine PGT screening beyond aneuploidies has value. Additionally, some incidental findings led to identification of a benign variant or a variant of uncertain significance which may cause patients undo anxiety, time, and cost for parental testing. While it was not possible to obtain detailed records on patient attitudes, family histories, and outcomes, many patients did seek out genetic counseling services through our center. Generally, these patients expressed anxiety regarding the findings. However, they also expressed an understanding of why these findings were being noted and an interest in determining the significance to the health of their embryos. While it is appropriate to be concerned regarding the impact that incidental findings may have on the emotional well-being of our patients, it is also important that we do not withhold potentially relevant information regarding the health of a patient’s embryos. In the context of prenatal testing, McGillivray et al. argue that withholding incidental findings from a prenatal CMA prevents patient autonomy and decision-making which could cause more harm to the parents of a future (child) [11]. We argue that patients should be informed of an incidental finding identified via PGT and offered the opportunity to pursue additional testing.

### Supplementary Information

Below is the link to the electronic supplementary material.

Supplementary file1 (XLSX 12 KB)

## Data Availability

Data is available upon request to the corresponding author.
